# Handbills for the Cure of Diseases!

**Published:** 1849-04

**Authors:** 


*Handbills for the cure of diseases!*—The following resolutions of the city authorities of New Orleans, relative to the posting of handbills for the sale of patent medicines and the cure of diseases, we copy from the New Orleans Medical and Surgical Journal:—

Council of Municipality, No. One,
Extract of the Sitting of Monday, 24th June, 1846.

*Be it further Resolved,* That the fact of announcing publicly, by posting hand-bills in public places, the sale of medicines for the cure of diseases, shall constitute a police misdemeanor.

*Be it further Resolved,* That a fine of twenty-five dollars shall be imposed on every bill sticker convicted of having posted up in one or several places, one or several hand-bills, offering for sale medicines for the cure of diseases.

[Signed] PAUL BERTUS, President,

A true copy: A. D. CROSSMAN, Mayor.

We think, with our contemporary, that the Council is entitled to credit for this action, and we should be glad to see the authorities of other cities follow so excellent an example. One cannot turn a corner of the streets, without the eye being greeted with such advertisements, many of which are grossly indelicate. It is a nuisance, which regard for decency requires should be abated. We know not how it is in New Orleans, but, in this city, the impertinence of those engaged in the business does not stop at posting advertisements,—agents being employed to distribute cards and pamphlets, by leaving them at the doors of private houses. Frequently the objects of such publications are disguised under the nominal appearance of almanacs or newspapers. The imposition upon the credulous, and thus obtaining money upon false pretences, is by no means the chief evil of these documents, but they are often filled with gross and disgusting details, peculiarly unfitted for the perusal of females and children, and doubtless conducive to immorality. The reform, to be complete, should not stop at handbills and pamphlets, but should extend to family newspapers, and even religious periodicals. We hope the time will arrive when the moral sense of the community will demand the entire expurgation of such things from the latter.

